# Substantia nigra Smad3 signaling deficiency: relevance to aging and Parkinson’s disease and roles of microglia, proinflammatory factors, and MAPK

**DOI:** 10.1186/s12974-020-02023-9

**Published:** 2020-11-16

**Authors:** Ying Liu, Lijia Yu, Yaling Xu, Xiaohui Tang, Xijin Wang

**Affiliations:** Department of Neurology, Xinhua Hospital Affiliated to Shanghai Jiao Tong University School of Medicine, 1665 Kongjiang Road, Shanghai, 200092 P.R. China

**Keywords:** Parkinson’s disease, Smad3 signaling, Aging, Microglia, Neuroinflammation, MAPK

## Abstract

**Background:**

Smad3 signaling is indicated to regulate microglia activity. Parkinson’s disease (PD) neurodegeneration is shown to be associated with aging and neuroinflammation. However, it remains unclear about the relationship among Smad3 signaling, aging, neuroinflammation, and PD.

**Methods:**

Rats were treated with SIS3 (a specific inhibitor of Smad3, intranigal injection) and/or lipopolysaccharide (intraperitoneal injection). We investigated the effect of SIS3 and lipopolysaccharide and their mechanism of action on motor behavior and nigrostriatal dopaminergic system in the rats. Furthermore, we explored the effect of SIS3 and LPS and their potential signaling mechanism of action on inflammatory response by using primary microglial cultures. Finally, we investigated the relationship among aging, Smad3 signaling, and neuroinflammation using animals of different ages.

**Results:**

Both SIS3 and lipopolysaccharide induced significant behavior deficits and nigrostriatal dopaminergic neurodegeneration in the rats compared with the vehicle-treated (control) rats. Significantly increased behavior deficits and nigrostriatal dopaminergic neurodegeneration were observed in the rats co-treated with SIS3 and lipopolysaccharide compared with the rats treated with vehicle, SIS3, or lipopolysaccharide. Furthermore, both SIS3 and lipopolysaccharide induced significant microglia activation and proinflammatory factor (IL-1β, IL-6, iNOS, and ROS) level increase in the SN of rats compared with the control rats. Significantly enhanced microglial inflammatory response was observed in the rats co-treated with SIS3 and lipopolysaccharide compared with the other three groups. For our in vitro study, both SIS3 and lipopolysaccharide induced significant proinflammatory factor level increase in primary microglia cultures compared with the control cultures. Significantly increased inflammatory response was observed in the cultures co-treated with SIS3 and lipopolysaccharide compared with the other three groups. MAPK (ERK/p38) contributed to microglial inflammatory response induced by co-treatment with SIS3 and lipopolysaccharide. Interestingly, there was decrease in Smad3 and pSmad3 expression (protein) and enhancement of neuroinflammation in the mouse SN with aging. Proinflammatory factor levels were significantly inversely correlated with Smad3 and pSmad3 expression.

**Conclusion:**

Our study strongly indicates the involvement of SN Smad3 signaling deficiency in aging and PD neurodegeneration and provides a novel molecular mechanism underlying the participation of aging in PD and helps to elucidate the mechanisms for the combined effect of multiple factors in PD.

## Introduction

Parkinson’s disease (PD), a common aging-related neurodegenerative disorder, is clinically characterized by resting tremor, bradykinesia, rigidity, and postural instability. The pathological features of PD are progressive degeneration of dopaminergic neurons in the substantia nigra (SN) and loss of dopamine terminals in the striatum [1, 2]. Aging is a primary risk factor for the etiopathogenesis of PD [3]. Epidemiological studies have shown that the prevalence in PD rises with increasing age [4]. To date, the etiopathogenesis of PD has not been fully elucidated. There are increasing evidences indicating that the etiology and pathogenesis of PD are multifactorial [5–8].

Neuroinflammation is widely believed to be involved in the etiopathogenesis of PD [9–11]. Microglia, the macrophage-like resident immune cells of the central nervous system (CNS), are shown to play a crucial role in nigrostriatal dopaminergic neurodegeneration by releasing proinflammatory factors such as interleukin-1β (IL-1β), interleukin-6 (IL-6), nitric oxide (NO), and reactive oxygen species (ROS) [9, 12, 13]. Microglia-derived proinflammatory factors are demonstrated to induce dopaminergic neuronal loss and further induce inflammatory cells to gather to the disease lesions, aggravating PD dopaminergic neurodegeneration [14, 15].

Smad3 is a main effector in the canonical signaling of transforming growth factor (TGF)-β1 [16]. Canonical TGF-β1 signal transduction pathway is comprised of ligand binding to the type II receptor (TβR-II) and subsequently phosphorylating the type I receptor (TβR-I). Upon phosphorylation, the receptor complex leads to the recruitment and phosphorylation of Smad2 and Smad3, which form a heteromeric complex with Smad4 and translocate into nucleus to regulate the expression of targeted genes [17, 18]. Smad3 signaling has been shown to be required for the development and homeostasis of microglia and plays a key role in the regulation of microglial activity [19]. Accumulating studies have indicated that Smad3 pathway participates in the anti-inflammatory property of TGF-β1 in the brain by inhibiting microglia activation and proinflammatory/immune response [20–25]. However, it remains unclear about the relationship among Smad3 signaling, aging, neuroinflammation, and PD.

In this study, rats were treated with SIS3 (intranigal injection, 4 μg/each side), a specific inhibitor of Smad3, and/or lipopolysaccharide (LPS, intraperitoneal injection, 1 mg/kg). We investigated the effect of SIS3 and LPS and their mechanism of action on motor behavior and nigrostriatal dopaminergic system in the rats. Furthermore, we explored the effect of SIS3 and LPS and their potential signaling mechanism of action on inflammatory response by using primary microglial cell cultures. Finally, considering that aging is one of the strongest risk factors for PD, we investigated the relationship among aging, Smad3 signaling, and neuroinflammation in the SN using animals of different ages.

## Materials and methods

### Drugs and reagents

The SIS3 (Sigma-Aldrich, St. Louis, MO, USA), a specific Smad3 phosphorylation inhibitor, has been shown to reduce the anti-inflammatory effect of TGF-β1 on microglia activation and inflammatory response [21]. Monoclonal antibodies against Smad3 and pSmad3 were from Cell Signaling Technology (Cambridge, MA, USA) [26]. Rabbit anti-TGF-β1 was from ABclonal (Wuhan, China) [27]. Polyclonal antibodies against ionized calcium-binding adaptor molecule-1 (IBA-1) and tyrosine hydroxylase (TH) were obtained from Abcam (USA) [28]. Monoclonal glial fibrillary acidic protein (GFAP) antibody was from Cell Signaling Technology (Cambridge, MA, USA) [29]. Polyclonal antibodies against phosphorylated p38 (p-p38) MAPK, p38 MAPK, phosphorylated ERK (p-ERK) MAPK, ERK MAPK, phosphorylated JNK (p-JNK), and JNK MAPK were from Cell Signaling Technology (Cambridge, MA, USA) [30]. Polyclonal antibodies against IL-1β and IL-6 were from ABclonal (Wuhan, China) [31]. Monoclonal antibodies against iNOS were from Sigma-Aldrich (St. Louis, MO, USA) [32]. PD98059 (ERK inhibitor), SB203580 (p38 inhibitor), and SP600125 (JNK inhibitor) were from Sigma-Aldrich (St. Louis, MO, USA). Secondary antibodies were purchased from Santa Cruz Biotechnology (Santa Cruz, CA, USA) [33]. Dimethyl sulfoxide (DMSO) and LPS (*Escherichia coli*, O111:B4) were purchased from Sigma-Aldrich (St. Louis, MO, USA). TGF-β1 was obtained from R&D Systems (USA).

### Animals and treatments

Male Sprague-Dawley rats (6–8 weeks) and male C57BL/6 mice (8–10 weeks) were purchased from Shanghai SLAG Laboratory Animal Corporation (Shanghai, China). The rats and mice were maintained in a room with temperature of 22 ± 1° C and relative humidity 50–60% under controlled lighting condition of 12/12-h cycle. All animals had free access to water and food. All procedures were conducted in accordance with the guidelines and regulations of the National Institutes of Health and were approved by the Ethics Committee of Xinhua Hospital affiliated to Shanghai Jiao Tong University School of Medicine.

For this study, considering feasibility and easy operation, based on the fact that rats are bigger than mice, we used rats in stereotaxical experiments to investigate the effect of SIS3 and LPS and mechanism of action on motor behavior and nigrostriatal dopaminergic system instead of mice. Rats were randomly divided into various experimental groups (vehicle group: *N* = 14, SIS3 group: *N* = 15, LPS group: *N* = 14, SIS3 + LPS group: *N* = 17). The rats received SIS3 or DMSO via a stereotaxic injection as follows [34, 35]: SIS3 (4 μg in 5 μl of DMSO) was stereotaxically injected into the SN on the right side of rats (5.5 mm posterior, 1.5 mm lateral, and 8.3 mm ventral to the bregma) over a period of 5 min. The injection needle was left in situ for another 5 min to avoid backflow. Similarly, rats were stereotaxically treated with SIS3 (4 μg/5 μl) on the other side of SN. Each rat received a total of 8 μg/10 μl of SIS3 administration. The vehicle-treated (control) rats were stereotaxically injected with equivalent volume (5 μl/each side) of DMSO. On the next day, the rats were treated with LPS or vehicle. For LPS administration, LPS was dissolved in saline at a concentration of 0.25 mg/ml. Based on the body weight of rats (250–300 g), 1.0–1.2 ml of LPS (1 mg/kg body weight) or equivalent volume of vehicle (saline) was administrated via single intraperitoneal (i.p.) injection. These rats were sacrificed by cervical dislocation on the 21st day after the last drug administration for subsequent experiments. Usually, aging animals are difficult to obtain for researches. Luckily, aging mice could be available at the time that we needed aging animals. Therefore, to investigate the relationship among aging, Smad3 signaling, and neuroinflammation in the SN, mice of different ages (young group 2–3 months, *N* = 6; aged group 18–20 months, *N* = 6) were used in our experiments. Detailed experimental processes and number of animals used for experiments are shown in Additional file 1: Table S1.

### Behavioral tests

#### Open field test

The behavioral tests were performed for three rounds (on the 7th, 14th, and 21st day after the last intraperitoneal injection of LPS) on the rats. To determine spontaneous locomotor activity, the rats were tested in the open field which consisted of a square plastic box (100 × 100 × 50 cm) with the inside walls painted black. Each rat was placed in the center of the box to freely explore for 5 min. The video recorded the movement and behavior of rats, and then, the SuperMaze V2.0 software (Shanghai Xin Ruan, China) was used to quantify three parameters: total distance, inactive sitting, and number of rearing.

#### Beam walk

Motor balance and coordination of rats were measured by beam walk test. Rats were consecutively trained to adapt the beam for 2 days before the first round of test. Rats were placed on a wood beam about 80-cm long and 1-cm wide suspended at 40-cm height and were allowed to cross the balance beam. Then, beam walk test was carried out on the 7th, 14th, and 21st day after the last drug administration. Three trials were performed on each rat, and the time that rats crossed the balance beam was recorded. Inter-trial interval was at least 1 h, and the time of three trials was averaged per rat.

#### Catalepsy test

The catalepsy test consisted of vertical grids and horizontal bars. For grid test, the limbs of rats were gently placed on a vertical grid (25.5-cm wide, 44-cm high, and 1-cm grid), and timing started. The timing stopped when the paws of rats were removed from the grid. For bar test, the front paws of rats were gently placed on a metal bar 9 cm above the floor. The timing stopped when the paws of rats climbed down from the bar. The duration was recorded as descent latency with a maximum of 120 s in both grid and bar tests.

### Primary microglial cell cultures

Primary microglial cell cultures were prepared from the cortical tissues of postnatal days 2–3 SD rat pups as previously described [36]. Briefly, the cortices of rat pups were dissected, removed the meninges and blood vessels, suspended in Hank’s Balanced Salt Solution (HBSS) supplemented with newborn calf serum, and digested with 0.25% EDTA trypsin for 15 min. After terminating digestion, the single cell suspension was centrifuged at 1000 rpm for 5 min and resuspended in Dulbecco’s modified Eagle’s medium (DMEM)/Ham’s F12 containing 10% heat-inactivated fetal bovine serum (FBS), penicillin (100 U/ml), and streptomycin (100 μg/ml). Then, cells were cultured in 75-cm^2^ flasks at 37 °C with a humidified atmosphere of 5% CO_2_. The medium was changed for the first time after 4 days. On days 10–14, microglia and astrocytes can be separated by shaking the flask for 1 h at 250 rpm. The enriched microglia were cultured at a density of 1 × 10^6^ cells/well in 6-well plates or 2 × 10^5^ cells/well in 24-well plates in DMEM/F12 supplemented with 10% FBS and 1 mM sodium pyruvate at 37 °C and 5% CO_2_. Above 95% of microglia was observed to be immunopositive for IBA-1, a marker for microglia [37].

### Drug exposures in vitro

To investigate the effect of SIS3 and LPS on inflammatory response in microglia, SIS3 (2 μl in 6-well plates or 1 μl in 24-well plates) or vehicle (DMSO) was added to the primary microglial cell cultures after enriched microglia were cultured for 1 day [38]. The final concentration of SIS3 was 10 μM. After 1 h of SIS3 incubation, the microglia were exposed to LPS (2 μl in 6-well plates or 1 μl in 24-well plates) or vehicle (phosphate-buffered saline, PBS). The final concentration of LPS was 300 ng/ml. TGF-β1 (2 μl in 6-well plates or 1 μl in 24-well plates) was applied to the microglia cultures (the final concentration was 10 ng/ml) 1 h following LPS or PBS, which were incubated for 24 h and then collected for subsequent experiments. It was particularly important that TGF-β1 was added to all groups in order to be sufficient to activate Smad3 signaling. To investigate the role of MAPK pathways in microglial inflammatory response, primary microglial cell cultures were treated with ERK inhibitor PD98059 (10 μM), p38 inhibitor SB203580 (10 μM), or JNK inhibitor SP600125 (10 μM) for 1 h as previously described [33]. Then, we applied SIS3 (10 μM) for 1 h followed by LPS (300 ng/ml) to microglia cultures with or without pre-treatment with inhibitors of MAPK pathways. Finally, for all groups, TGF-β1 (10 ng/ml) was applied for 24 h after 1 h of LPS administration.

### Immunohistochemistry

Perfusion of rats and immunostaining of brain sections were performed as previously described [30]. Briefly, rats were anesthetized and fixed with 4% paraformaldehyde in 0.1 M PBS by heart perfusion. The brain tissues were removed and placed in 4% paraformaldehyde overnight at 4 °C. Fixed tissues were embedded with paraffin and coronally cut with a microtome. According to the Paxinos and Watson rat brain atlas [39], a series of brain coronal sections (5-μm thickness) of the SN [from − 4.8 to – 5.8 mm relative to Bregma] and striatum [from 0.0 to + 1.0 mm relative to Bregma] were obtained from each animal. Sections at 30 μm intervals of the SN (total 5 sections per SN) and striatum (total 5 sections per striatum) were used for immunostaining. The brain sections were deparaffinized and rehydrated, and antigens were retrieved. After washing three times with PBS for 5 min each time, the sections were blocked with 3% bovine serum albumin (BSA) at room temperature for 30 min. Primary antibodies against TH (1:500), GFAP (1:1000), or IBA-1 (1:500) in 1% BSA in PBS were used to incubate overnight at 4 °C. These sections were washed three times and then incubated with secondary antibody in 1% BSA in PBS for 50 min at room temperature. Following washing three times, diaminobenzidine was used for coloration. Immunostained sections were viewed with a microscope (Olympus, Tokyo, Japan). For quantification of DA neurons, microglia, and astrocytes, as described previously [40], TH^+^ neurons and GFAP^+^ cells from the SN region and IBA-1^+^ cells from the SN and striatum regions were counted under a microscope with × 20 magnification by two independent and professional investigators blind to the treatment. The results were analyzed from the average. Five sections per rat were used to quantify the cell number. In the striatum, TH^+^ innervations were analyzed by optical density of TH-staining slices and measured by using the ImageJ software (NIH) under a microscope with × 20 magnification. Five sections per rat were used to quantify the optical density of TH^+^ innervations. Data was expressed as optical density of TH^+^ innervations (% of vehicle).

### Western blotting

Western blotting was performed as previously described [41–43]. The substantia nigra, striatum, and cerebellum tissues of rats or primary cultured microglia were homogenized in lysis buffer. Proteins were extracted from homogenized tissues or microglia and quantified to equal concentrations by using the BCA protein assay kit (Beyotime Biotechnology, China). The protein samples (40 μg) were separated by sodium dodecyl sulfate-polyacrylamide gels (SDS-PAGE) (Beyotime Biotechnology, China) and transferred onto PVDF membranes (Millipore, Billerica, MA, USA). The membranes were blocked with 5% not-fat milk in TBST for 1 h at room temperature, and subsequently incubated with primary antibodies against p-Smad3 (1:1000), Smad3 (1:1000), TGF-β1 (1:1000), ERK1/2 (1:1000), p-ERK1/2 (1:1000), p38 MAPK (1:1000), p-p38 MAPK (1:1000), JNK (1:1000), p-JNK (1:1000), iNOS (1:1000), IL-6 (1:1000), IL-1β (1:1000), or GAPDH (1:2000) overnight at 4 °C. The membranes were washed three times and incubated with HRP-labeled secondary antibodies (1:3000) for 1 h at room temperature. The protein bands were detected using a ECL assay kit and measured by a ChemiDoc^TM^ XRS+ imaging system (Bio-Rad Laboratories Inc., Hercules, CA, USA). The intensity of protein bands was measured by the Image J software (National Institutes of Health, NIH). Data are presented as a relative optical density normalized to GAPDH. For the analysis of MAPK pathways, the relative density was expressed as a ratio of the density of phosphoprotein to that of corresponding total protein.

### Reactive oxygen species measurement

As previously described [34, 44, 45], intracellular ROS production was measured by using the 2′-7′-dichloro-dihydro-fluorescein diacetate (DCFH-DA) assay kit (Sigma-Aldrich) based on the ROS-mediated conversion of DCFH-DA to fluorescent dichlorofluorescein (DCF). For in vivo detection, single cell suspensions were prepared from the substantia nigra homogenates of rats, and then incubated with DCFH-DA (the final concentration was 10 μM) for 20 min at 37 °C. The single cell suspensions were inverted every 3–5 min to ensure sufficient mixing and washed three times with PBS. Fluorescence microplate readers were used to detect fluorescence intensity at an excitation wavelength of 488 nm and an emission wavelength of 525 nm. For in vitro detection, culture medium of primary microglia was first removed. The microglia were washed with PBS and incubated with DCFH-DA (dissolved in fresh serum-free DMEM at a final concentration of 10 μM) for 20 min at 37 °C. Fluorescence intensity was measured by using fluorescence microplate readers at 488/525 nm.

### Reverse transcription-quantitative polymerase chain reaction

Total RNA was extracted from substantia nigra tissues of rats by using TRIzol reagent, and RT-qPCR was performed as previously described [46]. Total RNA was quantified using the Nanodrop-2000 (Thermo Fisher Scientific) and reverse transcribed into complementary DNA (cDNA) using a PrimeScript RT Reagent kit. According to the manufacturer’s instructions, real-time PCR analysis was performed with a SYBR green PCR kit in the ABI PRISM 7500 Sequence Detection system (Thermo Fisher Scientific Inc.). Three independent experiments were carried out on each sample. The levels of targeted mRNAs were determined with a comparative threshold cycle (Ct) method. GAPDH was used as the internal control.

The primer sequences used in the real-time quantitative PCR analysis were as follows: rat GAPDH (F: ATGATTCTACCCACGGCAAG; R: CTGGAAGATGGTGATGGGTT); rat IL-6 (F: CGAGCCCACCAGGAACGAAAGTC; R: CTGGCTGGAAGTCTCTTGCGGAG); rat IL-1β (F: TGCTGATGTACCAGTTGGGG; R: CTCCATGAGCTTTGTACAAG); rat iNOS (F: TGTGCTAATGCGGAAGGTCAT; R: CGACTTTCCTGTCTCAGTAGCAAA); rat TNF-α (F: TACTCCCAGGTTCTCTTCAA; R: CCAGGCTGACTTTCTCCTGG); mouse GAPDH (F: CTTTGTCAAGCTCATTTCCTGG; R: TCTTGCTCAGTGTCCTTGC); mouse IL-6 (F: TAGTCCTTCCTACCCCAATTTCC, R: TTGGTCCTTAGCCACTCCTTC); mouse IL-1β (F: GCAACTGTTCCTGAACTCAACT, R: ATCTTTTGGGGTCCGTCAACT); mouse iNOS (F: ATGTCCGAAGCAAACATCAC, R: TAATGTCCAGGAAGTAGGTG).

### Statistical analyses

The data are expressed as mean ± SEM. The ImageJ software (NIH) was used for densitometry determination in western blotting analysis. Student’s *t* test was used for comparisons between two groups and analysis of variance (ANOVA) followed by Bonferroni post hoc test for comparisons among various groups. Pearson value was reported in correlation analysis. All statistical analyses were performed using SPSS 17.0 (SPSS Inc., Chicago, USA) software. Values of *p* < 0.05 were considered to be statistically significant.

## Results

### Effect of SIS3 and LPS on motor behavior in rats

**Fig. 1:**
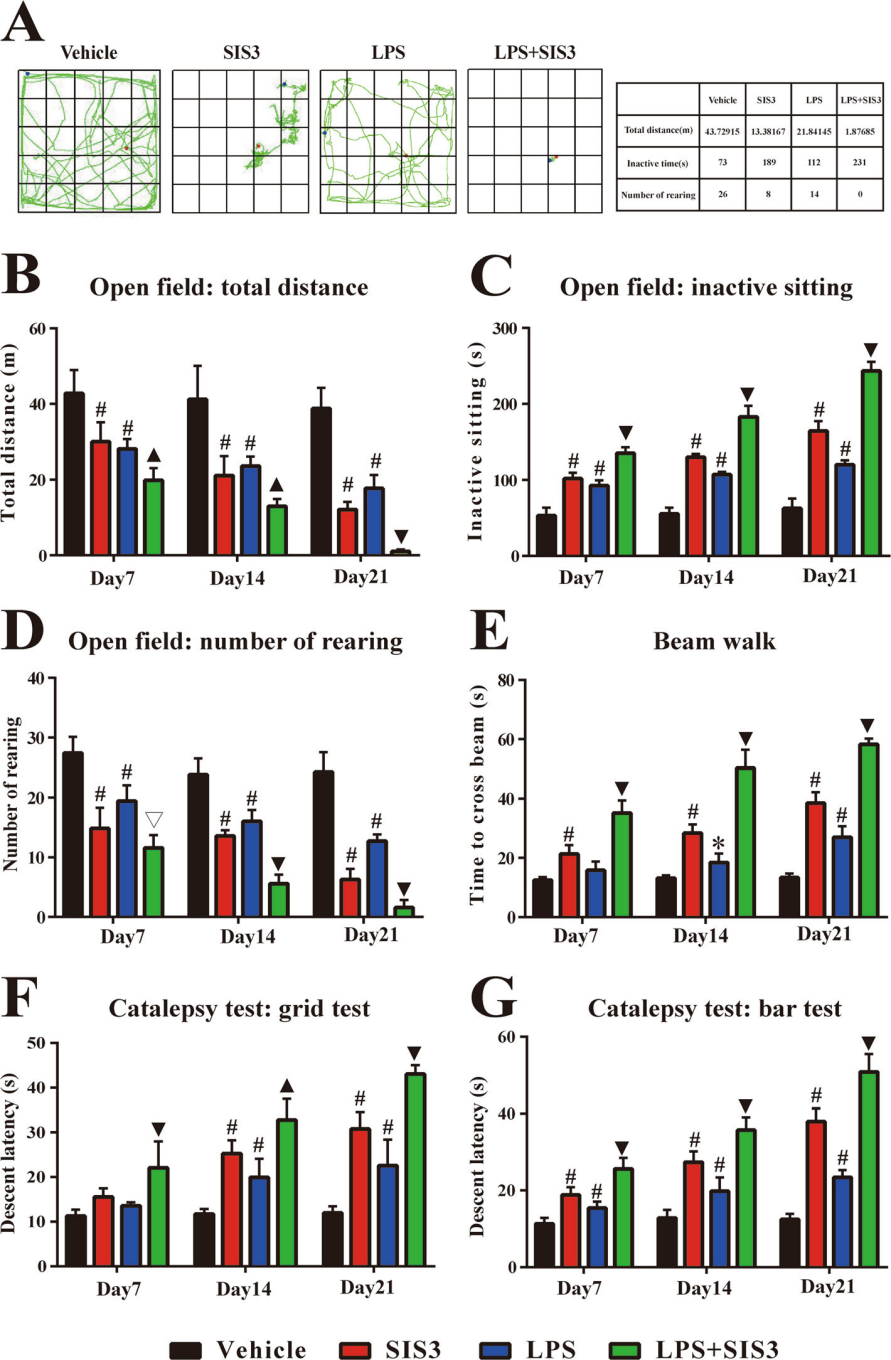
Effect of SIS3 and LPS on motor behavior of rats in behavioral tests. **a** Representative movement paths of different groups in open field test on the 21st day after the last drug administration. The corresponding values of total distance, inactive time and rearing number are shown in the right table. **b** Total distance traveled in open field test. **c** Inactive time in open field test. **d** Number of rearing (both front paws of rats off the ground with or without forepaws against a wall) in open field test. **e** Time to cross beam in beam walk test. **f** Descent latency in grid test. **g** Descent latency in bar test. Results are expressed as mean ± SEM. *N* = 7. *p < 0.05, compared with the rats treated with vehicle; #*p* < 0.01, compared with the rats treated with vehicle; ▽*p* < 0.01, compared with the rats treated with vehicle or LPS; ▲*p* < 0.05, compared with the rats treated with vehicle, SIS3 or LPS; ▼*p* < 0.01, compared with the rats treated with vehicle, SIS3, or LPS. LPS, lipopolysaccharide (1 mg/kg); SIS3 (4 μg/each side)

As shown in Additional file 2: Table S2, there was no significant difference in body weight of rats among the four groups before treatment. Also, there was no significant difference in body weight of rats among the four groups after treatment (Additional file 2: Table S2). To investigate the effect of SIS3 and LPS on rat behavior, behavioral tests including open field, beam walk, and catalepsy tests were performed on rats on the 7th, 14th, and 21st day after the last drug administration. On the three rounds of open field test, both SIS3 and LPS significantly decreased total distance and rearing number and increased inactive sitting time in the rats compared with the vehicle-treated (control) rats (*p* < 0.01, Fig. 1a–d). Significantly decreased total distance (days 7 and 14: *p* < 0.05, day 21: *p* < 0.01) and increased inactive sitting time (*p* < 0.01) were observed in the rats co-treated with SIS3 and LPS compared with the rats treated with vehicle, SIS3, or LPS (Fig. 1a–c). In addition, SIS3 and LPS co-treatment significantly decreased rearing number in the rats on the 7th day after the last drug administration compared with the rats treated with vehicle or LPS (*p* < 0.01) and significantly decreased it on the 14th and 21st day compared with the rats treated with vehicle, SIS3, or LPS (*p* < 0.01, Fig. 1d). The representative movement paths in open field test on the 21st day after the last drug administration are shown in Fig. 1a. The corresponding values of total distance, inactive time, and rearing number are shown in the right table of Fig. 1a. In summary, rats treated with SIS3 or LPS alone or simultaneously presented less movement around the arena and preferred to stay still compared with the control rats, which was reflected by decreased total distance as shown in Fig. 1b and by increased inactive sitting time as shown in Fig. 1c. In addition, reduced vertical locomotor activity was observed in the rats treated with SIS3 or LPS alone or simultaneously compared with the control rats, which was reflected by decreased number of rearing (both front paws of rats off the ground with or without forepaws against a wall) as shown in Fig. 1d. Correspondingly, as shown in Fig. 1e, on the three rounds of beam walk test, SIS3 significantly increased beam walk time in the rats compared with the control rats (*p* < 0.01). LPS significantly increased beam walk time in the rats on the 14th day (*p* < 0.05) and 21st day (*p* < 0.01) but did not significantly increase it on the 7th day compared with the control rats. Significantly increased beam walk time was observed in the rats co-treated with SIS3 and LPS compared with the other three groups (*p* < 0.01). For catalepsy test, as shown in Fig. 1f and g, on the 7th day, both SIS3 and LPS did not significantly increase descent latency of grid test whereas significantly increased that of bar test (*p* < 0.01) in the rats compared with the control rats. On the 14th and 21st day, both SIS3 and LPS significantly increased descent latency of grid and bar test in the rats compared with the control rats (*p* < 0.01). In addition, on the three rounds of the test, significantly increased descent latency of grid (day 14: *p* < 0.05, days 7 and 21: *p* < 0.01) and bar (*p* < 0.01) tests were observed in the rats co-treated with SIS3 and LPS compared with the other three groups. These results showed that both SIS3 and LPS induced behavior deficits in the rats, including reduced spontaneous locomotor activity and motor balance with stiff limbs. Moreover, SIS3 and LPS displayed a cumulative effect when simultaneously administrated to rats.

### Effect of SIS3 and LPS on nigrostriatal dopaminergic system in rats

**Fig. 2:**
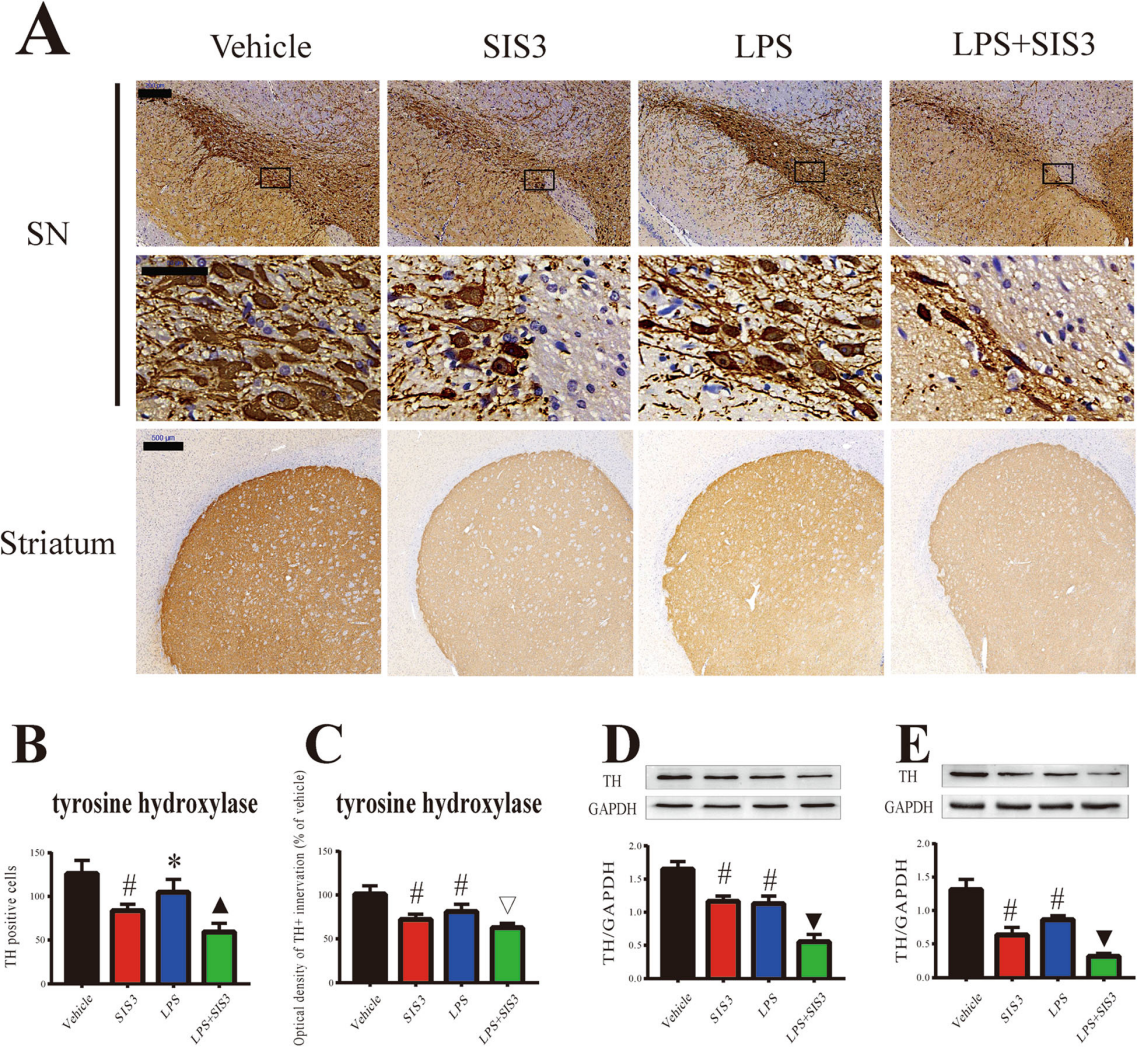
Effect of SIS3 and LPS on nigrostriatal dopaminergic system in rats. **a** Representative figures of TH expression (immunohistochemistry) in the SN and striatum. Scale bar = 200 μm in the SN (upper panels); 50 μm in the SN (below panels); and 500 μm in the striatum. **b** Quantification of TH^+^ neuron number in the SN is shown. **c** Quantification of TH^+^ innervations in the striatum is shown. **d** Protein level of TH in the SN was detected by western blotting and its quantitative analysis. **e** Protein level of TH in the striatum was detected by western blotting and its quantitative analysis. The results are expressed as mean ± SEM. *N* = 6. *p < 0.05, compared with the rats treated with vehicle; #*p* < 0.01, compared with the rats treated with vehicle; ▽*p* < 0.01, compared with the rats treated with vehicle or LPS; ▲*p* < 0.05, compared with the rats treated with vehicle, SIS3, or LPS; ▼*p* < 0.01, compared with the rats treated with vehicle, SIS3, or LPS. LPS, lipopolysaccharide (1 mg/kg); SIS3 (4 μg/each side)

To investigate the effect of SIS3 and LPS on nigrostriatal dopaminergic system of rats, TH immunopositive (TH^+^) neurons and innervations and the expression of TH protein in the SN and striatum of rats were evaluated by using immunohistochemistry and western blotting, respectively. As shown in Fig. 2a and b, both SIS3 (*p* < 0.01) and LPS (*p* < 0.05) significantly reduced the number of TH^+^ neurons in the SN of rats compared with the control rats. Significantly reduced number of TH^+^ neurons was observed in the SN of rats co-treated with SIS3 and LPS compared with the rats treated with vehicle, LPS, or SIS3 (*p* < 0.05). In addition, both SIS3 and LPS significantly reduced TH expression in the SN of rats compared with the control rats (*p* < 0.01, Fig. 2d). Significantly reduced TH expression was observed in the SN of rats co-treated with SIS3 and LPS compared with the other three groups (*p* < 0.01, Fig. 2d).

In the striatum, both SIS3 and LPS significantly decreased striatal TH^+^ innervations in the rats compared with the control rats (*p* < 0.01, Fig. 2a, c). Significantly decreased TH^+^ innervations were observed in the striatum of rats co-treated with SIS3 and LPS compared with the rats treated with vehicle or LPS (*p* < 0.01, Fig. 2a, c). As shown in Fig. 2e, both SIS3 and LPS significantly decreased TH protein expression in the striatum of rats compared with the control rats (*p* < 0.01). Significantly decreased TH expression was observed in the striatum of rats co-treated with SIS3 and LPS compared with the other three groups (*p* < 0.01).

### Effect of SIS3 and LPS on microglial activity and proinflammatory factors in rats

**Fig. 3:**
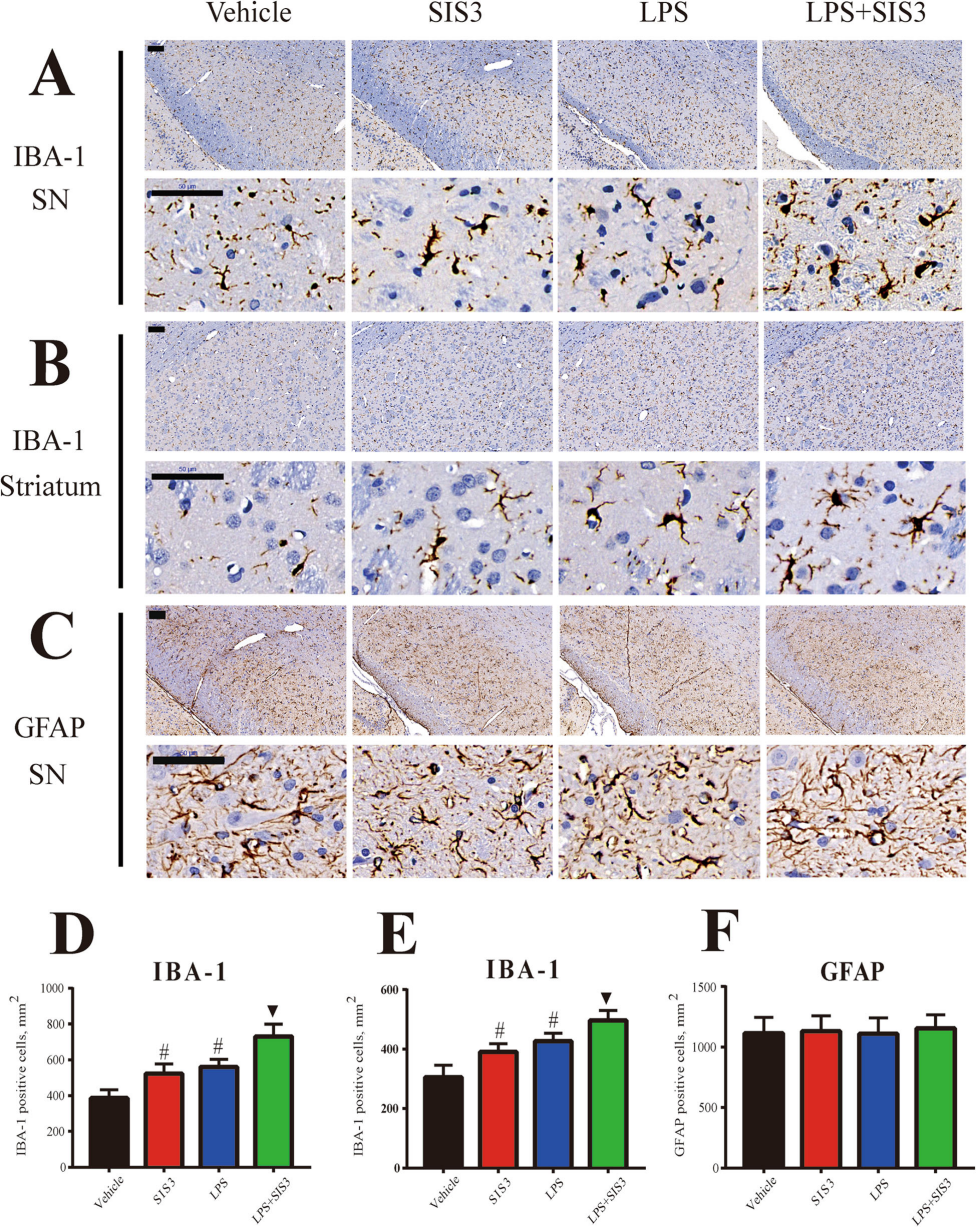
Effect of SIS3 and LPS on IBA-1 and GFAP expression in rats. **a** Representative figures of microglia and astrocyte (immunohistochemistry of IBA-1 and GFAP) in the SN and striatum. Scale bar = 100 μm in the upper panels of **a**, **b,** and **c**; 50 μm in the below panels of **a**, **b,** and **c**. **d** Quantification of IBA-1^+^ cell number in the SN is shown. **e** Quantification of IBA-1^+^ cell number in the striatum is shown. **f** Quantification of GFAP^+^ cell number in the SN is shown. Results are expressed as mean ± SEM. *N* = 6. #*p* < 0.01, compared with the rats treated with vehicle; ▼*p* < 0.01, compared with the rats treated with vehicle, SIS3, or LPS. LPS, lipopolysaccharide (1 mg/kg); SIS3 (4 μg/each side)

To investigate the effect of SIS3 and LPS on neuroinflammation, we assessed microglial and astrocytic responses in the SN of rats. As shown in Fig. 3a and d, both SIS3 and LPS significantly increased the number of IBA-1^+^ cells in the rat SN compared with the control rats (*p* < 0.01). Significantly increased number of IBA-1^+^ cells was observed in the SN of rats co-treated with SIS3 and LPS compared with the rats treated with vehicle, LPS, or SIS3 (*p* < 0.01). Furthermore, both SIS3 and LPS significantly increased the number of IBA-1^+^ cells in the rat striatum compared with the control rats (*p* < 0.01, Fig. 3b, e). Significantly increased number of IBA-1^+^ cells was observed in the striatum of rats co-treated with SIS3 and LPS compared with the other three groups (*p* < 0.01, Fig. 3b, e). Morphologically, IBA-1^+^ cells seemed to have more enlarged cell bodies in the SIS3, LPS, and LPS + SIS3 groups compared with the vehicle group (Fig. 3a, b). However, no significant difference in GFAP^+^ cell number was observed in the SN of rats among four groups (Fig. 3c, f).

**Fig. 4:**
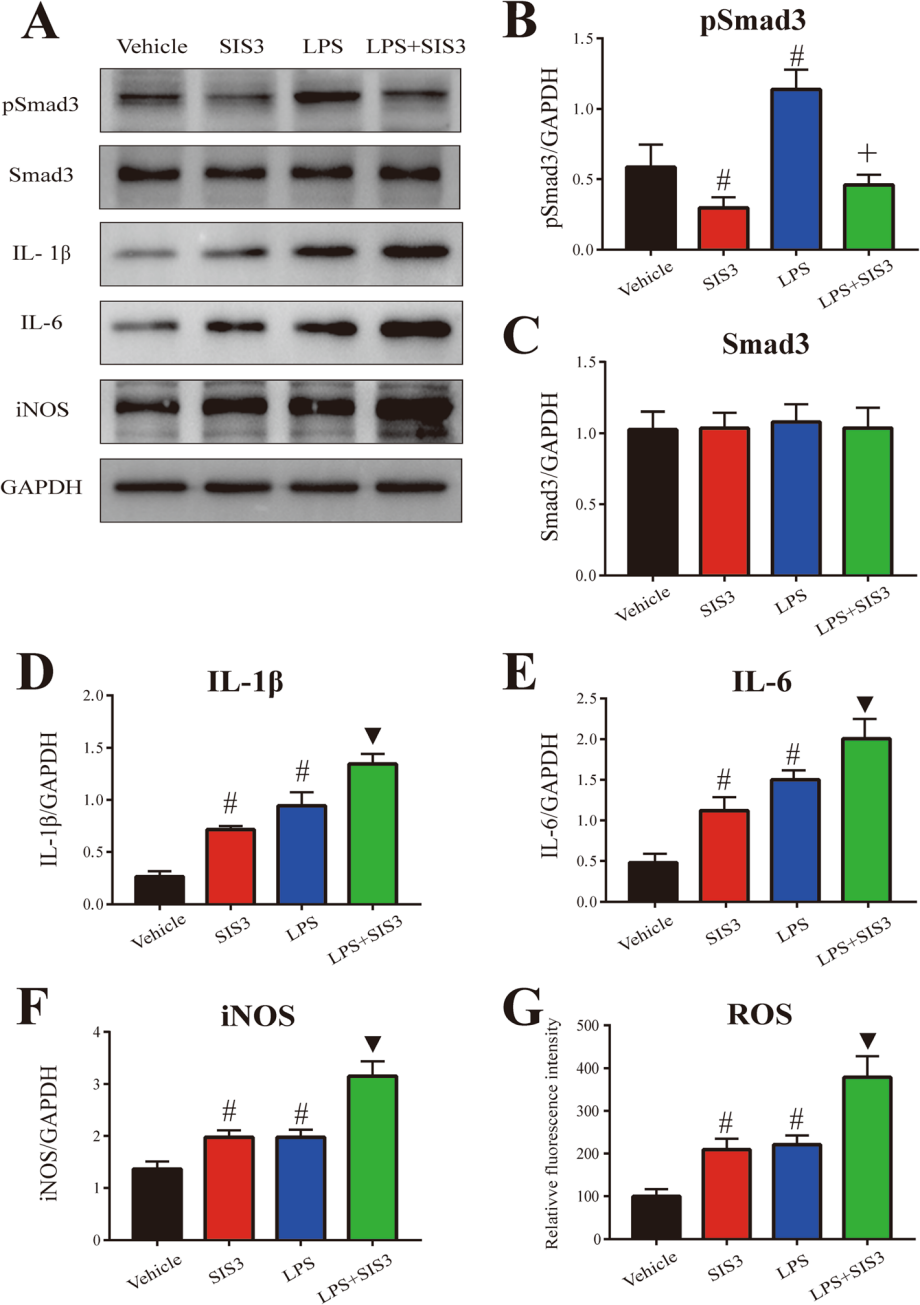
Effect of SIS3 and LPS on proinflammatory factors in rats. **a** Representative figures of Smad3, pSmad3, and proinflammatory factor expression (western blotting). **b**–**f** Histogram represents quantitation of pSmad3 (**b**), Smad3 (**c**), IL-1β (**d**), IL-6 (**e**), and iNOS (**f**) normalized to corresponding GAPDH. **g** ROS production was determined by DCFHDA. Results are expressed as mean ± SEM. *N* = 6. #*p* < 0.01, compared with the rats treated with vehicle; ▼*p* < 0.01, compared with the rats treated with vehicle, SIS3, or LPS; +*p* < 0.05, compared with the rats treated with SIS3, or LPS. LPS, lipopolysaccharide (1 mg/kg); SIS3 (4 μg/each side)

In accordance with microglial response, as shown in Fig. 4a and d–g, both SIS3 and LPS significantly enhanced proinflammatory factor (IL-1β, IL-6, iNOS, and ROS) levels in the SN of rats compared with the control rats (*p* < 0.01). Significantly enhanced levels of IL-1β, IL-6, iNOS, and ROS were observed in the SN of rats co-treated with SIS3 and LPS compared with the other three groups (*p* < 0.01).

As shown in Fig. 4a–c, consistent with the previous study [47], no significant Smad3 expression change was observed in the SN of rats treated with SIS3 compared with the control rats. As expected, SIS3 significantly decreased the expression of pSmad3 in the rat SN compared with the control rats (*p* < 0.01). Interestingly, LPS did not significantly increase the expression of Smad3 but significantly increased pSmad3 expression (*p* < 0.01) in the SN of rats compared with the control rats. SIS3 and LPS co-treatment significantly increased pSmad3 protein level in the SN of rats compared with the rats treated with SIS3, and significantly reduced pSmad3 expression in the SN of rats compared with the rats treated with LPS (*p* < 0.05). There was no significant Smad3 expression change in the SN of rats co-treated with SIS3 and LPS compared with the other three groups. In addition, as shown in Additional file 3: Figure S1, LPS significantly increased the expression of TGF-β1 in the rat SN compared with the control rats (*p* < 0.01). However, no significant TGF-β1 expression change was observed in the SN of rats treated with SIS3 compared with the control rats. SIS3 and LPS co-treatment significantly increased TGF-β1 expression in the SN of rats compared with the rats treated with vehicle or SIS3 (*p* < 0.01).

### Effect of SIS3 and LPS on proinflammatory factors in microglia cultures

**Fig. 5:**
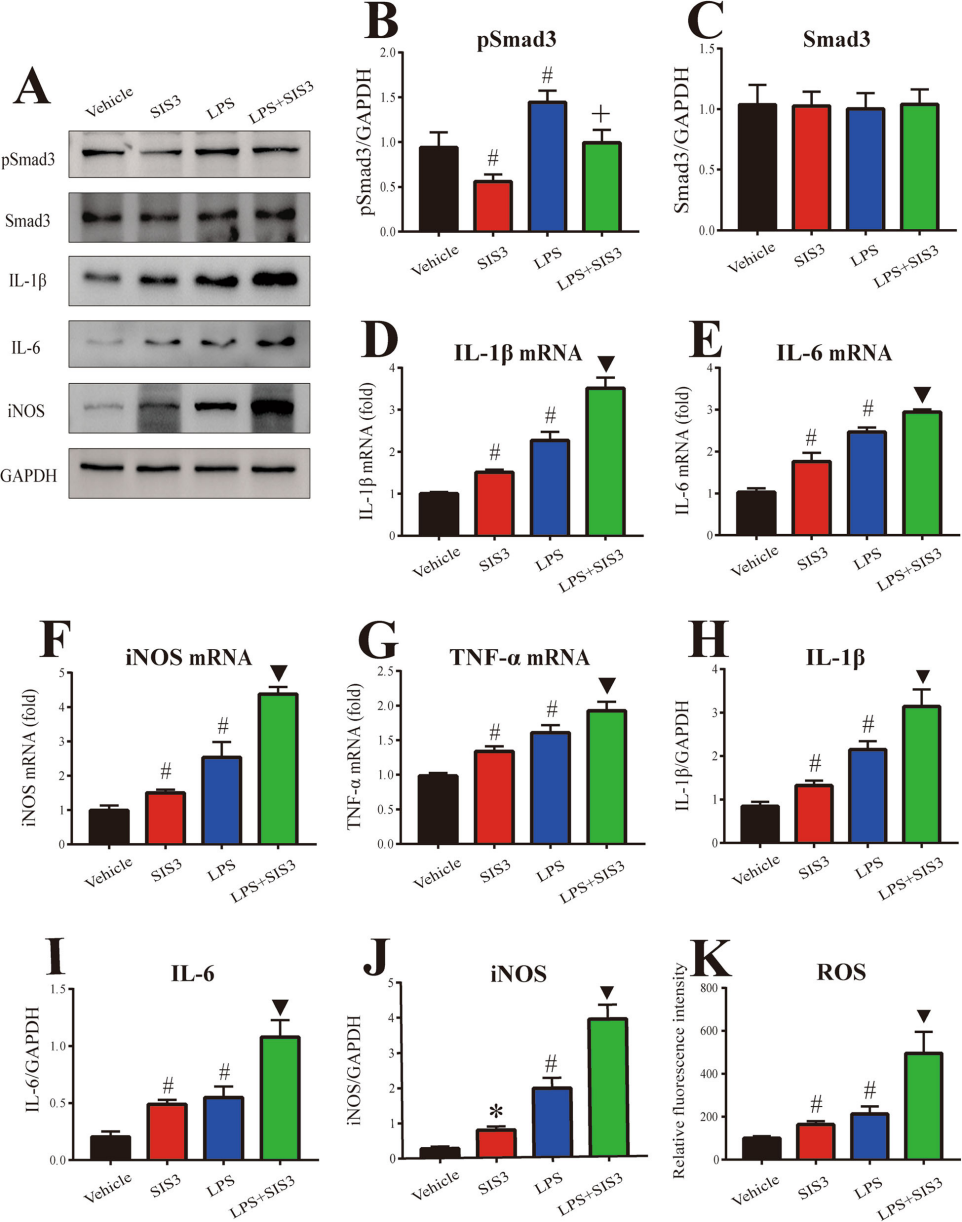
Effect of SIS3 and LPS on proinflammatory factors in microglia cultures. **a** Representative figures of Smad3, pSmad3, and proinflammatory factor expression (western blotting). **b**, **c** Histogram represents quantitation of pSmad3 (**b**) and Smad3 (**c**) normalized to corresponding GAPDH. **d**–**g** mRNA levels of IL-1β (**d**), IL-6 (**e**), iNOS (**f**), and TNF-α (**g**) were detected by RT-qPCR. **h**–**j** Histogram represents quantitation of IL-1β (**h**), IL-6 (**i**), and iNOS (**j**) normalized to corresponding GAPDH. (**k**) ROS production was determined by DCFHDA. It was particularly important to add TGF-β1 to all groups. Results are expressed as mean ± SEM. *N* = 6. *p < 0.05, compared with the cultures treated with vehicle; #*p* < 0.01, compared with the cultures treated with vehicle; ▼*p* < 0.01, compared with the cultures treated with vehicle, SIS3, or LPS; +*p* < 0.05, compared with the cultures treated with SIS3 or LPS. LPS, lipopolysaccharide (300 ng/ml); SIS3 (10 μM)

To further determine the effect of SIS3 and LPS on microglia-mediated inflammatory response, we assessed Smad3, pSmad3, and TGF-β1 expression and proinflammatory factor levels in primary microglia cultures. As shown in Fig. 5a–c, no significant difference in Smad3 expression was observed in microglia among four groups. In addition, SIS3 significantly decreased the level of pSmad3 protein (*p* < 0.01), but LPS significantly increased pSmad3 protein expression (*p* < 0.01) in the primary microglia cultures compared with the vehicle-treated cultures. SIS3 and LPS co-treatment significantly increased pSmad3 expression in the microglia cultures compared with the cultures treated with SIS3, and significantly reduced the expression of pSmad3 in the microglia cultures compared with the cultures treated with LPS (*p* < 0.05). As shown in Additional file 4: Figure S2, LPS significantly increased the expression of TGF-β1 (*p* < 0.01), but SIS3 did not significantly increase it in the primary microglia cultures compared with the control cultures. Significantly increased expression of TGF-β1 was observed in the microglia cultures co-treated with SIS3 and LPS compared with the cultures treated with vehicle or SIS3 (*p* < 0.01).

In accordance with the in vivo results, both SIS3 and LPS significantly enhanced proinflammatory factor (IL-1β, IL-6, iNOS, and TNF-α) mRNA levels in the microglia cultures compared with the control cultures (*p* < 0.01, Fig. 5d–g). Significantly enhanced mRNA levels of IL-1β, IL-6, iNOS, and TNF-α were observed in the cultures co-treated with SIS3 and LPS compared with the other three groups (*p* < 0.01, Fig. 5d–g). Furthermore, as shown in Fig. 5a and h–k, both SIS3 and LPS significantly enhanced the protein expression of IL-1β (*p* < 0.01), IL-6 (*p* < 0.01), and iNOS (SIS3: *p* < 0.05, LPS: *p* < 0.01), and ROS level (*p* < 0.01) in the microglia cultures compared with the control cultures. Significantly enhanced protein expression of IL-1β, IL-6, and iNOS and ROS level were observed in the cultures co-treated with SIS3 and LPS compared with the other three groups (*p* < 0.01).

### Role of MAPK pathways in microglial inflammatory response induced by SIS3 and LPS co-treatment

**Fig. 6:**
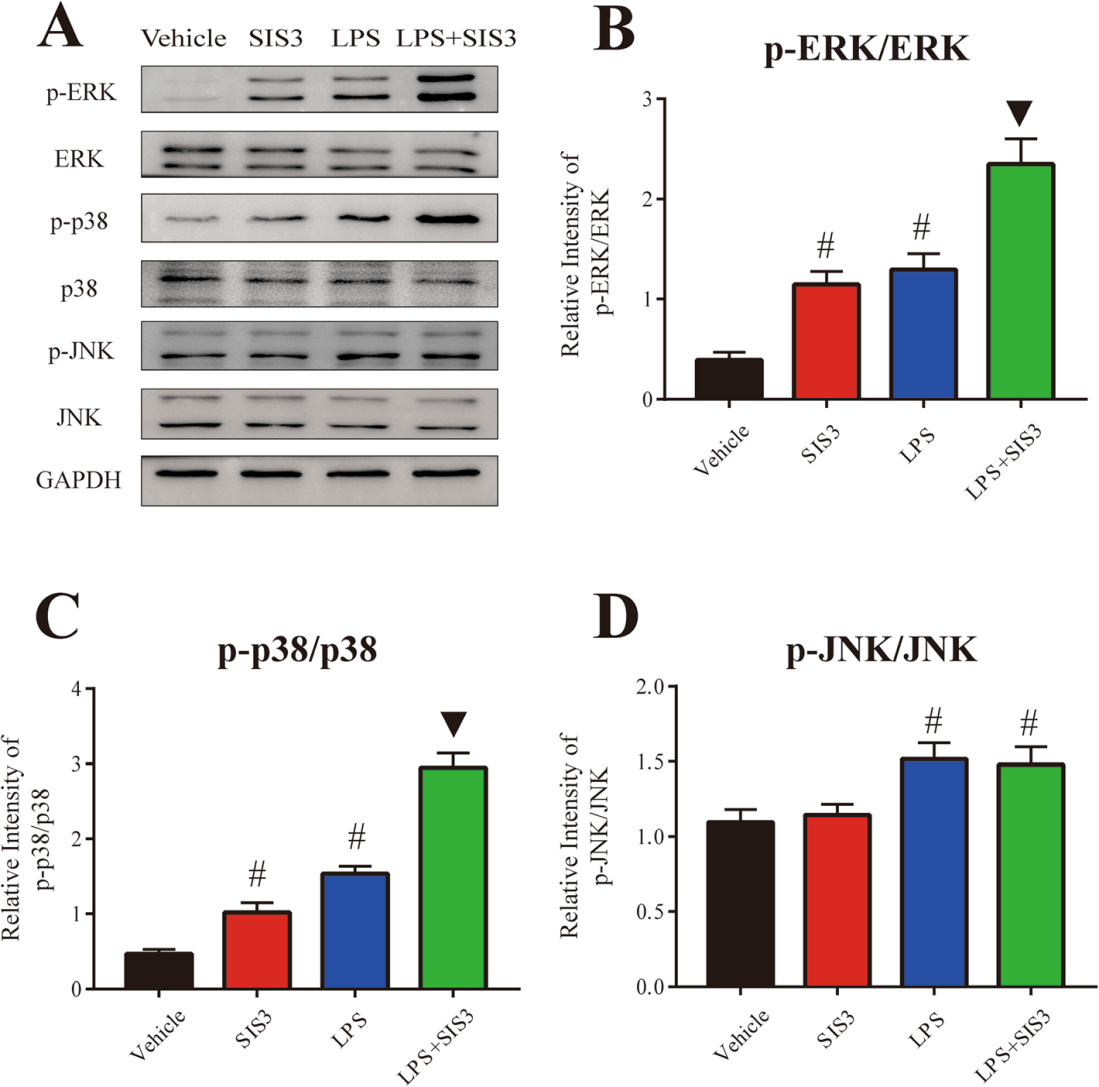
Effect of SIS3 and LPS on MAPK pathways in microglia cultures. **a** Representative figures of ERK, p38, and JNK phosphorylation (p-ERK, p-p38, and p-JNK) expression and total ERK, p38, and JNK expression (western blotting). **b**–**d** Histogram represents the ratio of p-ERK to total ERK (**b**), p-p38 to total p38 (**c**), and p-JNK to total JNK (**d**). It was particularly important to add TGF-β1 to all groups. Results are expressed as mean ± SEM. *N* = 6. #*p* < 0.01, compared with the cultures treated with vehicle; ▼*p* < 0.01, compared with the cultures treated with vehicle, SIS3, or LPS. LPS, lipopolysaccharide (300 ng/ml); SIS3 (10 μM)

Previous studies have suggested that MAPK pathways, including ERK, p38, and JNK, play a crucial role in the regulation of glia activation and inflammation [48]. To determine whether microglial inflammatory response induced by SIS3 and LPS were mediated by activation of MAPK pathways, we assessed the phosphorylation of ERK, p38, and JNK in microglia. As shown in Fig. 6a–c, both SIS3 and LPS significantly induced the phosphorylation of ERK and p38 in the microglia cultures compared with the control cultures (*p* < 0.01). However, although LPS significantly induced the phosphorylation of JNK (*p* < 0.01), SIS3 did not significantly induce JNK phosphorylation in the microglia cultures compared with the control cultures (Fig. 6d). Significantly enhanced phosphorylation of ERK and p38 was observed in the cultures co-treated with SIS3 and LPS compared with the other three groups (*p* < 0.01, Fig. 6a–c). As shown in Fig. 6d, although SIS3 and LPS co-treatment significantly induced the phosphorylation of JNK in the cultures compared with the control cultures (*p* < 0.01), there was no difference in JNK phosphorylation between LPS and LPS + SIS3 groups, suggesting that SIS3 treatment might not aggravate LPS-induced activation of JNK in microglia.

**Fig. 7:**
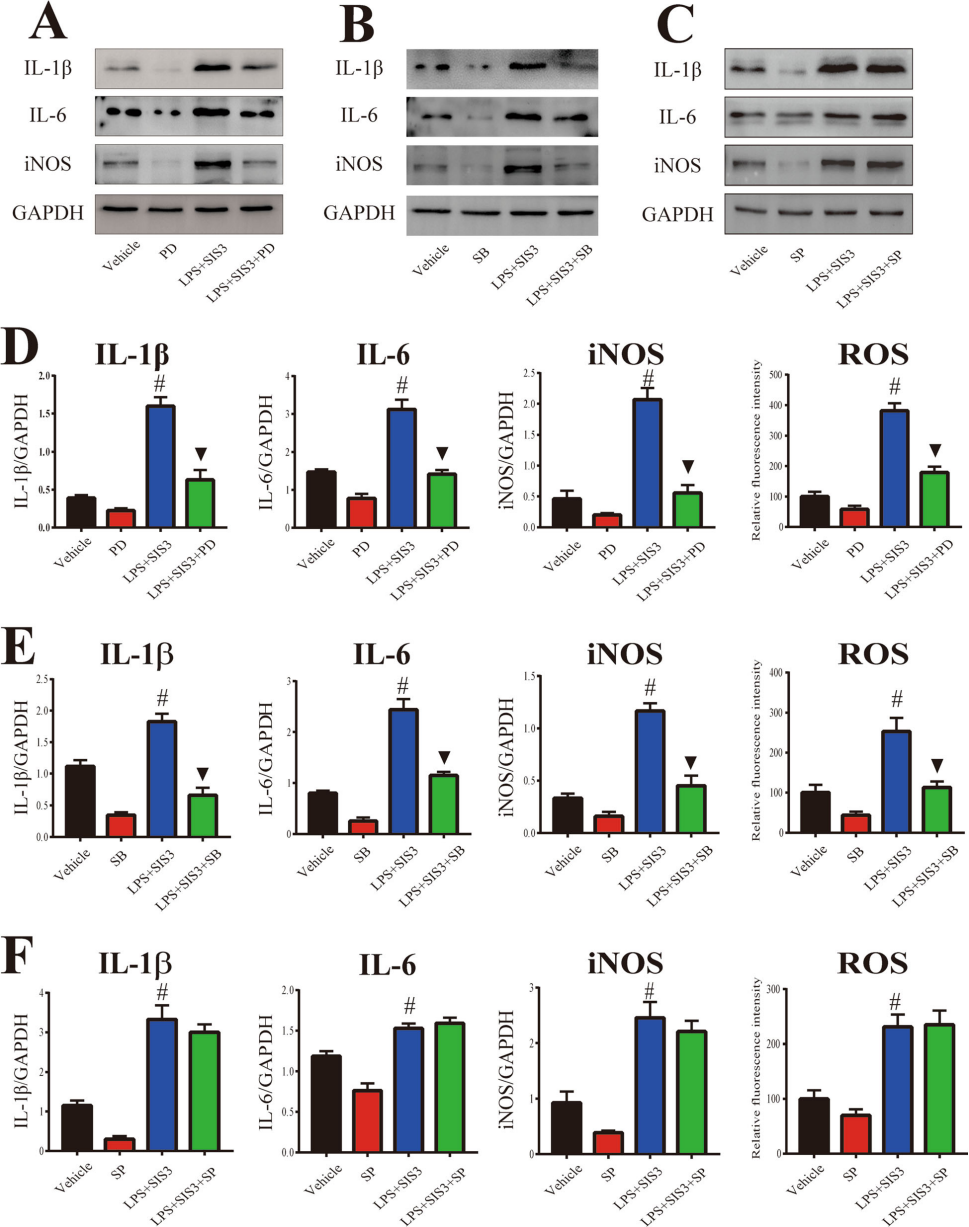
Role of MAPK pathways in microglial inflammatory response induced by SIS3 and LPS co-treatment. **a**–**c** Representative figures of proinflammatory factor expression (western blotting) in microglia cultures pretreated with PD (**a**), SB (**b**), and SP (**c**). **d**–**e** Histogram represents quantitation of IL-1β, IL-6, and iNOS normalized to corresponding GAPDH. ROS production was determined with DCFHDA. It was particularly important to add TGF-β1 to all groups. Results are expressed as mean ± SEM. *N* = 6. #*p* < 0.01, compared with the cultures treated with vehicle; ▼*p* < 0.01, compared with the cultures co-treated with SIS3 and LPS. LPS, lipopolysaccharide (300 ng/ml); SIS3 (10 μM); PD, PD980599, ERK inhibitor (10 μM); SB, SB203580, p38 inhibitor (10 μM). SP, SP600125, JNK inhibitor (10 μM)

Based on the above results, to further investigate the role of MAPK pathways in microglial inflammatory response induced by SIS3 and LPS co-treatment, microglia cultures were pretreated with the selective inhibitor of ERK (PD98059), p38 (SB203580), or JNK (SP600125) for 1 h. As shown in Fig. 7, the enhanced levels of proinflammatory factors (IL-1β, IL-6, iNOS, and ROS) in response to SIS3 and LPS co-treatment were significantly inhibited by preincubation with ERK inhibitor PD98059 and p38 inhibitor SB203580 in the microglia cultures (*p* < 0.01). Nevertheless, JNK inhibitor SP600125 had no significant effect on the increased proinflammatory factor levels induced by co-treatment with SIS3 and LPS in the cultures (Fig. 7c, f). These results indicate that microglial inflammatory response induced by co-treatment with SIS3 and LPS may be mediated by activation of ERK and p38 MAPK.

### Aging-related proinflammatory factor level enhancement and Smad3 and pSmad3 expression decrease in mouse SN

**Fig. 8:**
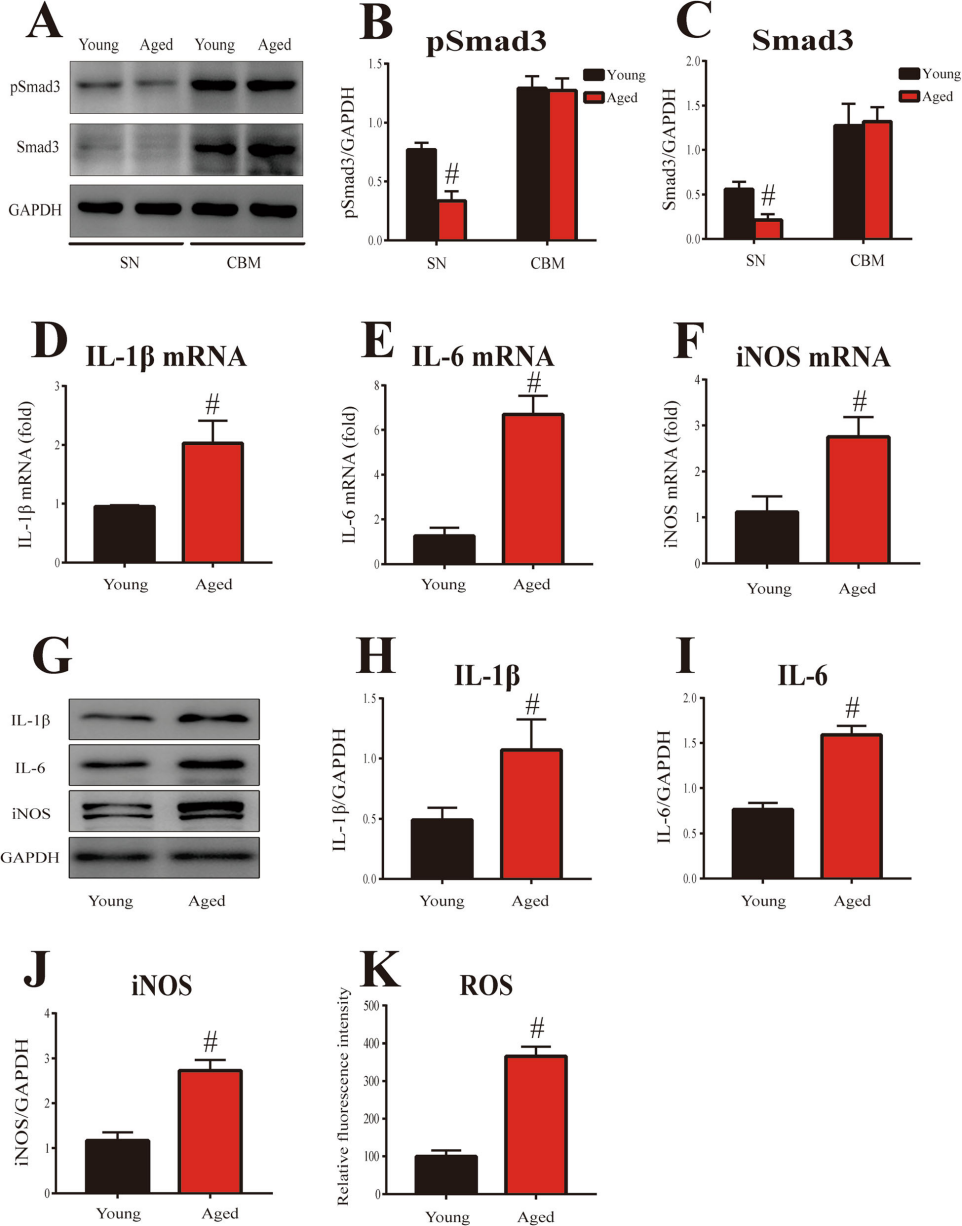
Aging-related Smad3 and pSmad3 expression and proinflammatory factor levels in mouse SN. **a** Representative figures of Smad3 and pSmad3 expression (western blotting) in the SN and cerebellum of mice. **b**–**c** Histogram represents quantitation of pSmad3 (**b**) and Smad3 (**c**) normalized to corresponding GAPDH. **d**–**f** mRNA levels of IL-1β (**d**), IL-6 (**e**), and iNOS (**f**) were detected by RT-qPCR in mouse SN. **g** Representative figures of proinflammatory factor expression (western blotting) in mouse SN. **h**–**j** Histogram represents quantitation of IL-1β (**h**), IL-6 (**i**), and iNOS (**j**) normalized to corresponding GAPDH. **k** ROS production was determined with DCFHDA. Results are expressed as mean ± SEM. *N* = 6. #*p* < 0.01, compared with the young mice. SN, substantia nigra; CBM, cerebellum

PD is an aging-related neurodegenerative disease. To identify whether neuroinflammatory response is increased with aging, we assessed the proinflammatory factor (IL-1β, IL-6, iNOS, and ROS) levels in the SN of young (8–10 weeks) and aged (18–20 months) mice. As shown in Fig. 8d–f, the mRNA levels of IL-1β, IL-6, and iNOS were significantly increased in the SN of aged mice compared with those of young mice (*p* < 0.01). Western blot analysis also showed that significant increase in IL-1β, IL-6, and iNOS protein expression was observed in the SN of aged mice compared with those of young mice (*p* < 0.01, Fig. 8g–j). In addition, the level of ROS was significantly increased in the SN of aged mice compared with those of young mice (*p* < 0.01, Fig. 8k).

**Fig. 9:**
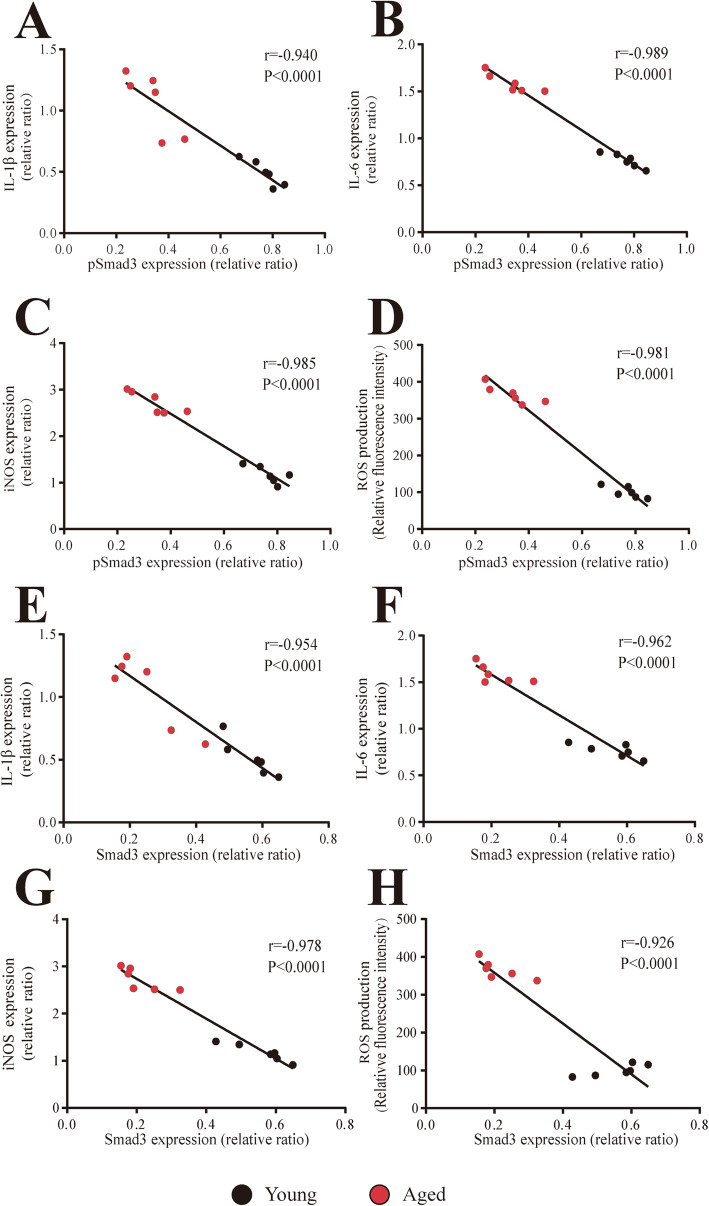
Correlation of proinflammatory factor levels with Smad3 and pSmad3 expression in mouse SN. **a**–**d** Significant inverse correlation of IL-1β (**a**), IL-6 (**b**), iNOS (**c**), and ROS (**d**) levels with pSmad3 expression in mouse SN. **e**–**h** Significant inverse correlation of IL-1β (**e**), IL-6 (**f**), iNOS (**g**), and ROS (**h**) levels with Smad3 expression in mouse SN. Aged mice: *N* = 6; young mice: *N* = 6

Interestingly, in contrast with the above inflammatory response enhancement, the protein levels of Smad3 and pSmad3 were significantly decreased in the SN of aged mice compared with those of young mice (*p* < 0.01, Fig. 8a–c). However, no significant difference in Smad3 and pSmad3 protein expression was observed in the cerebellum between young and aged mice. Furthermore, there was no significant TGF-β1 expression change in the SN of aged mice compared with the young mice (Additional file 5: Figure S3). Finally, correlation analysis showed proinflammatory factor (IL-1β: *r* = − 0.954; IL-6: *r* = − 0.962; iNOS: *r* = − 0.978; ROS: *r* = − 0.926) levels were significantly inversely correlated with Smad3 expression (*p* < 0.0001, Fig. 9e–h). In addition, significant inverse correlation was observed between proinflammatory factor (IL-1β: *r* = − 0.940; IL-6: *r* = − 0.989; iNOS: *r* = − 0.985; ROS: *r* = − 0.981) levels and pSmad3 expression (*p* < 0.0001, Fig. 9a–d).

## Discussion

Smad3, coupled to the TGF-β1 receptor, is a key canonical signaling molecule of TGF-β1 pathway. Smad3 signaling is indicated to regulate microglia activity. PD neurodegeneration is shown to be associated with aging and neuroinflammation. However, it remains unclear about the relationship among Smad3 signaling, aging, neuroinflammation, and PD.

SIS3 has been demonstrated to reduce TGF-β1-induced Smad3 phosphorylation and interaction of Smad3 with Smad4 [47]. As a specific inhibitor of Smad3, SIS3 is widely used to investigate the role of Smad3 signaling in many diseases. In this study, rats were stereotaxically administrated with SIS3 (4 μg/each side) and/or intraperitoneally injected with LPS (1 mg/kg). LPS has been shown to enter CNS through the blood-brain barrier to induce neuroinflammation and oxidative stress and reproduce the pathological features of human PD in rats [49]. We observed that not only LPS but also SIS3 induced significant behavior deficits and nigrostriatal dopaminergic neurodegeneration in the rats compared with the control rats. Moreover, SIS3 exacerbated LPS-induced behavior deficits and nigrostriatal dopaminergic neurodegeneration in the rats. These results reveal that Smad3 signaling deficiency may participate in PD neurodegeneration. In addition, in this study, behavioral tests were performed three rounds on rats on the 7th, 14th, and 21st day after the last drug administration. Rats treated with SIS3 or LPS alone or simultaneously presented progressively severe behavior deficits over time, suggesting that SIS3 or LPS alone or in combination induced disease progression in a time-dependent manner.

It has been widely accepted that microglia activation and inflammatory/immune response can damage neurons and are responsible for neurodegenerative diseases [50, 51]. As the main immune cells of CNS, microglia are the first line of defense [52]. Under resting conditions, microglia have essential functions of surveying environment, providing neuronal support, and promoting phagocytosis and clearance of cell debris [53]. However, over-activated microglia change the morphology of small cell bodies with ramified processes into enlarged cell bodies and shorten cellular processes [54]. Moreover, following activation, microglia change their functions and produce proinflammatory factors including inflammatory cytokines and reactive species, which contribute to neurotoxicity [55]. In this study, we showed that both SIS3 and LPS significantly induced microglia activation reflected by microglia number increase and morphological changes, as well as enhanced levels of IL-1β, IL-6, iNOS, and ROS in the SN of rats. We also observed that SIS3 was capable of significantly aggravating LPS-induced microglia activation and proinflammatory factor level increase in the SN of rats. Smad3 has been indicated to play an important role in regulation of immune response. Previous studies have shown that Smad3 deficiency promotes proinflammatory factor production in mast cells and macrophages treated with LPS [56, 57]. Impaired mucosal immune function was observed in mutant mice due to Smad3 disruption [58]. In addition, Smad3 pathway participates in the induction of quiescent microglial phenotype by TGF-β1 [22]. TGF-β1-Smad3 signaling modifies Aβ clearance through modulating microglial activity [23]. Recently, it has been shown that TGF-β1 inhibits microglial inflammatory response and exerts neuroprotective effect in a MPP^+^ rat model of PD via Smad3 pathway [21]. In this study, our results strongly indicate that Smad3 signaling deficiency-mediated microglial inflammatory response may be involved in PD dopaminergic neurodegeneration. However, in our study, SIS3 did not significantly induce astrocyte activation in the SN of rats with or without LPS administration. This result is not consistent with the previous study showing decreased number of astrocytes in the SN of Smad3 null mice [59]. In fact, Wang and his colleagues [60] have shown that Smad3 is necessary for the TGF-β1-mediated p15^INK4B^ induction and astrocyte growth inhibition by using astrocyte cultures derived from Smad3 null mice. In response to neuronal insults, the number of astrocytes may not be decreased but probably increase to a certain extent in Smad3 deficiency. Furthermore, SIS3 (a specific inhibitor of Smad3) was used in our study, which is different from the genetic method of Smad3 knock-out in other researches. The above result difference between our and the previous study may be attributed to different experimental conditions. It has been widely accepted that astrocytes have the capacity of trophic support to neurons [61]. Although astrocytes can regulate the immune reactivity in response to various stimuli, microglia have been suggested to display much more cytotoxic effects on dopaminergic neurons than astrocytes [62, 63]. On the one hand, there is different distribution of microglia throughout the brain regions with the highest microglial concentration in the SN [64, 65]. On the other hand, over-activated microglia release a large number of inflammatory cytokines such as IL-1β, IL-6, and TNFα and oxidation products such as NO and ROS [66]. Enhanced inflammatory cytokines further induce ROS production and oxidative stress [67]. In turn, increased oxidative stress reaction enhances proinflammatory factor production and amplifies inflammatory response, which results in a vicious circle of neuroinflammation and oxidative insults [15]. Dopaminergic neurons in the SN are particularly sensitive to oxidative damage due to their reduced antioxidant capacity [68]. Therefore, the enrichment of microglia in the SN combined with the intrinsic characteristics of dopaminergic neurons may be responsible for selective dopaminergic neurodegeneration mediated by microglial inflammatory response.

In the present study, we further investigated potential signaling mechanism underlying Smad3 signaling deficiency-mediated microglial inflammatory response through primary microglia cultures. We observed that LPS significantly increased proinflammatory factor levels in primary microglia cultures compared with the control cultures. Furthermore, SIS3 not only significantly enhanced the levels of proinflammatory factors but also significantly aggravated the LPS-induced proinflammatory factor level increase in microglia cultures, which are consistent with our in vivo results and further support the role of Smad3 signaling deficiency in PD neurodegeneration. MAPK pathways have been known to be involved in the induction of microglial proinflammatory factor production [48]. In agreement with the previous study [69], our results showed that LPS significantly increased the phosphorylation of ERK, p38, and JNK in the microglial cultures compared with the control cultures. SIS3 significantly induced the phosphorylation of ERK and p38, but not JNK, in the cultures. Also, SIS3 significantly enhanced the LPS-induced phosphorylation of ERK and p38, but not JNK, in the cultures. We further used the inhibitors of ERK (PD98059), p38 (SB203580), and JNK (SP600125) to determine whether MAPK activation is responsible for inflammatory response enhancement in microglia co-treated with SIS3 and LPS. We observed that SIS3-enhanced proinflammatory factor levels in LPS-treated microglia were significantly inhibited by ERK and p38 inhibitors, but not by JNK inhibitor. Therefore, our results indicate that ERK/p38 MAPK may be a contributor to Smad3 signaling deficiency-mediated microglial inflammatory response. Of course, we could not rule out the possibility of the involvement of other pathways including JNK MAPK. Consistent with our study, ERK and p38 inhibitors have been shown to reduce the expression of inflammatory cytokines, iNOS, and COX-2 induced by thrombin in cortical microglia cultures [70]. Other studies showed that suppression of JNK and ERK reduced LPS-mediated microglia activation in BV cells [71] and that inhibition of p38 signaling rescued dopaminergic neuron degeneration by inhibiting microglia polarization in a mouse model of PD [72]. All the above differences regarding the role of MAPK pathways in microglial regulation may be attributed to different experimental conditions.

As expected, in the present study, SIS3 significantly reduced pSmad3 expression, but not Smad3 expression in the rat SN and microglia cultures, which is consistent with the previous studies [47, 73]. Interestingly, we observed that LPS significantly increased pSmad3 protein level in the rat SN and microglia cultures, although there was no significant change in the level of Smad3 protein. Moreover, in the study, LPS significantly increased TGF-β1 expression in the rat SN and microglia cultures with or without SIS3 administration compared with the control group. As an anti-inflammatory regulator, TGF-β1, and thus TGF-β1/Smad3 signaling is predominantly upregulated under pathological inflammatory conditions to modulate and attenuate inflammation [74]. LPS, widely used as a proinflammatory stimuli, has been reported to increase the expression of TGF-β1 and its downstream signaling protein pSmad3 in primary microglia or hippocampus of young mice [26, 75], which is in agreement with our results.

Aging is a major risk factor for PD [3]. However, it is still unknown about the relationship among aging, Smad3 signaling, and neuroinflammation in the SN. In the present study, we observed that the levels of proinflammatory factors were increased in the SN of mice with aging by comparing mice of different ages. Interestingly, the expression of SN Smad3 and pSmad3 was decreased in normal aging. Proinflammatory factor levels were significantly inversely correlated with Smad3 and pSmad3 expression in the SN. These results indicate that SN Smad3 signaling deficiency may participate in the induction of aging-related neuroinflammation and ultimately promote the development and progression of PD. In addition, there was no significant difference in Smad3 and pSmad3 protein expression in the cerebellum between young and aged mice, suggesting a selective distribution of aging-related Smad3 signaling deficiency. Based on the regional selectivity of neuronal loss in PD [76], the above results further support the notion that Smad3 signaling deficiency may participate in PD dopaminergic neurodegeneration. From another point of view, our results provide a novel molecular mechanism underlying the participation of aging in PD. In aged brain, there is an elevated neuroinflammation characterized by senescent and over-responsive microglia and cumulative proinflammatory factors [67, 77]. Aging-related Smad3 signaling deficiency, at least partially, is a significant cause for the dysfunction of aged microglia in the SN. Therefore, aging-related Smad3 signaling impairment may contribute, at least partially, to the etiopathogenesis of PD. In addition to the property of inducing microglia activation, Smad3 signaling deficiency has been implicated to promote dopamine catabolism and α-synuclein aggregation in Smad3 null mice [59]. Further studies are needed to investigate precise mechanisms for the role of Smad3 signaling deficiency in aging and PD neurodegeneration.

## Conclusion

Our results show that SN Smad3 signaling deficiency may induce microglial inflammatory response mediated by ERK/p38 MAPK activation and ultimately result in nigrostriatal dopaminergic neurodegeneration. It is interesting that there were decrease in Smad3 and pSmad3 protein expression and enhancement of neuroinflammation in the mouse SN with aging. Proinflammatory factor levels were significantly inversely correlated with Smad3 and pSmad3 expression in the SN. Our results not only provide a new evidence for the involvement of SN Smad3 signaling deficiency in PD neurodegeneration, but also suggest a novel molecular mechanism underlying participation of aging in PD. In addition, our study helps to elucidate the mechanisms for the combined effect of multiple factors in PD.

## Supplementary Information

**Additional file 1: Table S1.** Detailed experimental processes and number of animals used for experiments.

**Additional file 2: Table S2.** Effect of each treatment on body weight and survival of rats.

**Additional file 3: Figure S1.** Effect of SIS3 and LPS on TGF-β1 expression in the SN of rats. (A) Representative figures of TGF-β1 expression (western blotting). (B) Histogram represents quantitation of TGF-β1 normalized to corresponding GAPDH. Results are expressed as mean ± SEM. N = 6. #p < 0.01, compared with the rats treated with vehicle; &p < 0.01, compared with the rats treated with vehicle or SIS3. LPS, Lipopolysaccharide (1 mg/kg); SIS3 (4 μg/each side).

**Additional file 4: Figure S2.** Effect of SIS3 and LPS on TGF-β1 expression in microglia cultures. (A) Representative figures of TGF-β1 expression (western blotting). (B) Histogram represents quantitation of TGF-β1 normalized to corresponding GAPDH. Results are expressed as mean ± SEM. N = 6. #p < 0.01, compared with the rats treated with vehicle; &p < 0.01, compared with the rats treated with vehicle or SIS3. LPS, Lipopolysaccharide (300 ng/ml); SIS3 (10 μM).

**Additional file 5: Figure S3.** TGF-β1 expression in the SN of young and aged mice. (A) Representative figures of TGF-β1 expression (western blotting). (B) Histogram represents quantitation of TGF-β1 normalized to corresponding GAPDH. Results are expressed as mean ± SEM. N = 6.

## Data Availability

The datasets generated and/or analyzed during the current study are available from the corresponding author on reasonable request.
