# Supplementary material for: Substantia nigra Smad3 signaling deficiency: relevance to aging and Parkinson’s disease and roles of microglia, proinflammatory factors, and MAPK
Source: J Neuroinflammation. 2020 Nov 16;17:342. doi: 10.1186/s12974-020-02023-9 (PMC7670688; doi:10.1186/s12974-020-02023-9)
Supplement: Supplementary file 1 — Additional file 1: Table S1. Detailed experimental processes and number of animals used for experiments. [file 12974_2020_2023_MOESM1_ESM.doc]

Additional file 1: Table S1. Detailed experimental processes and number of animals used for experiments.

| To investigated the effect of SIS3 and LPS and mechanism of action on motor behavior and nigrostriatal dopaminergic system in the rats | Phase I | Group | Behavior tests  (Day 7, 14, 21 after the last drug administration) | Immunohistochemistry  (Day 21 after the last drug administration) |
| --- | --- | --- | --- | --- |
|  |  | Vehicle  (N = 8) | N = 7  (One died after anesthetization) | N = 6  (Heart perfusion failure: N = 1) |
|  |  | SIS3  (N = 8) | N = 7  (One died after stereotaxic injection) | N = 6  (Tissue preparation failure: N = 1) |
|  |  | LPS  (N = 8) | N = 7  (One died after the last drug administration) | N = 6  (Heart perfusion failure: N = 1) |
|  |  | SIS3+LPS  (N = 9) | N = 7  (Two died after the last drug administration) | N = 6  (Heart perfusion failure: N = 1) |
|  | Phase II | Group | Western blotting and ROS measurement  (Day 21 after the last drug administration) | |
|  |  | Vehicle  (N = 6) | N = 6 | |
|  |  | SIS3  (N = 7) | N = 6  (One died after stereotaxic injection) | |
|  |  | LPS  (N = 6) | N = 6 | |
|  |  | SIS3+LPS  (N = 8) | N = 6  (One died after anesthetization; One died after the last drug administration) | |
| To investigated the relationship among aging, Smad3 signaling and neuroinflammation in the SN | | Group | Further experiments | |
|  |  | Young (N = 6) | Western blotting, ROS measurement and RT-qPCR | |
|  |  | Aged (N = 6) |  |  |

LPS, Lipopolysaccharide; SIS3, Specific inhibitor of Smad3; SN, Substantia nigra; ROS, Reactive oxygen species; RT-qPCR, Reverse transcription-quantitative polymerase chain reaction.
