# Supplementary material for: Substantia nigra Smad3 signaling deficiency: relevance to aging and Parkinson’s disease and roles of microglia, proinflammatory factors, and MAPK
Source: J Neuroinflammation. 2020 Nov 16;17:342. doi: 10.1186/s12974-020-02023-9 (PMC7670688; doi:10.1186/s12974-020-02023-9)
Supplement: Supplementary file 2 — Additional file 2: Table S2. Effect of each treatment on body weight and survival of rats. [file 12974_2020_2023_MOESM2_ESM.doc]

Additional file 2: Table S2. Effect of each treatment on body weight and survival of rats.

| Group | Treatment | Body weight (g)  (Day 0 before treatment) | Body weight (g)  (Day 21 after treatment) | Death rate  (%) |
| --- | --- | --- | --- | --- |
| Vehicle  (N = 14) | DMSO+saline | 268.9 ± 3.388 | 377.6 ± 4.866 | 7.14 |
| SIS3  (N = 15) | SIS3+saline | 270 ± 4.419 | 374.8 ± 3.873 | 13.33 |
| LPS  (N = 14) | DMSO+LPS | 267.5 ± 3.451 | 375.1 ± 3.06 | 7.14 |
| SIS3+LPS  (N = 17) | SIS3+LPS | 269.3 ± 3.612 | 372.5 ± 3.809 | 23.53 |

There was no significant difference in body weight of rats among four groups before treatment. Also, there was no significant difference in body weight of rats among four groups after treatment.
