# Supplementary figures and images for: Substantia nigra Smad3 signaling deficiency: relevance to aging and Parkinson’s disease and roles of microglia, proinflammatory factors, and MAPK
Source: J Neuroinflammation. 2020 Nov 16;17:342. doi: 10.1186/s12974-020-02023-9 (PMC7670688; doi:10.1186/s12974-020-02023-9)

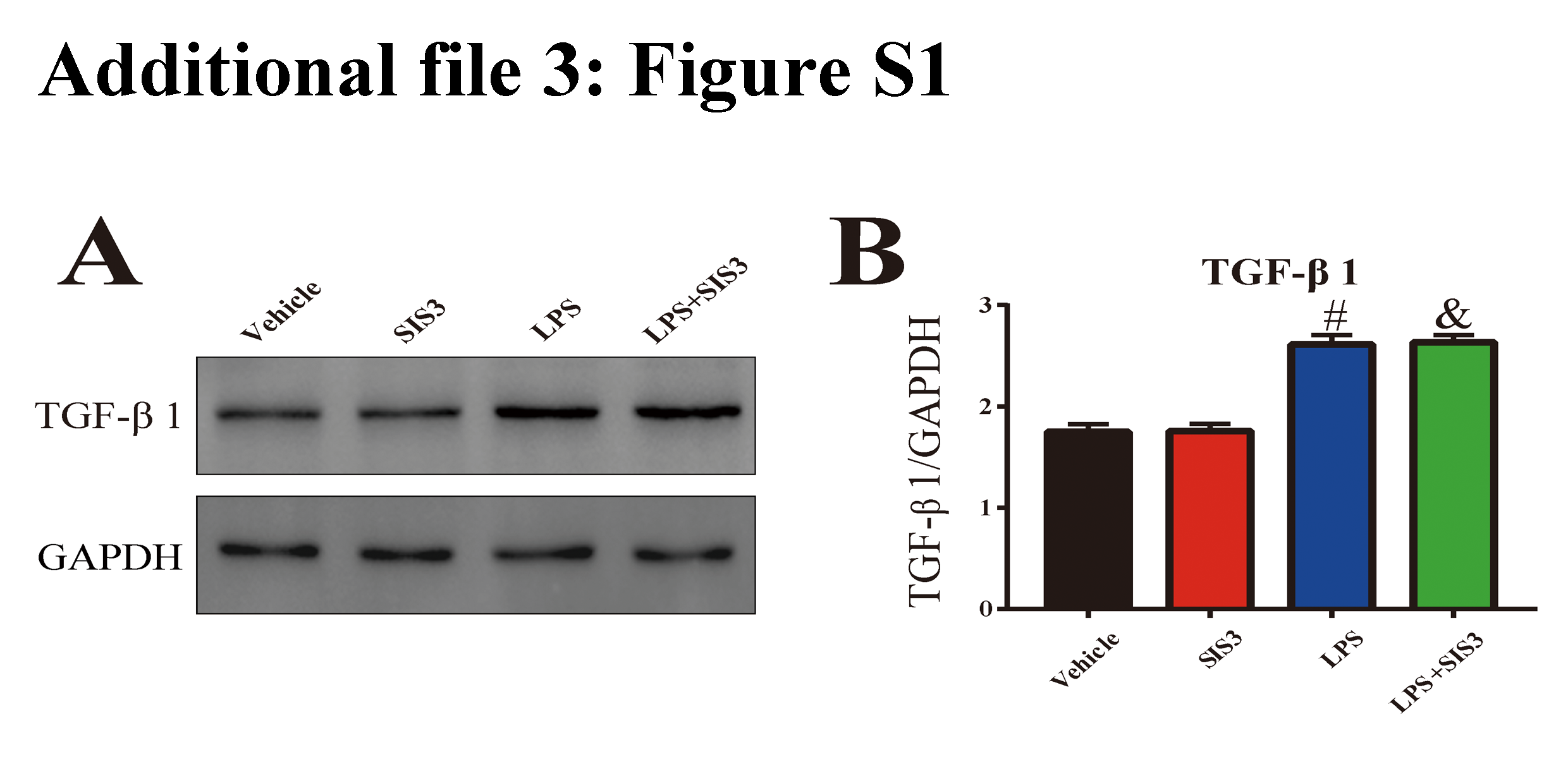

Supplement: Supplementary file 3 — Additional file 3: Figure S1. Effect of SIS3 and LPS on TGF-β1 expression in the SN of rats. (A) Representative figures of TGF-β1 expression (western blotting). (B) Histogram represents quantitation of TGF-β1 normalized to corresponding GAPDH. Results are expressed as mean ± SEM. N = 6. #p < 0.01, compared with the rats treated with vehicle; &p < 0.01, compared with the rats treated with vehicle or SIS3. LPS, Lipopolysaccharide (1 mg/kg); SIS3 (4 μg/each side). [file 12974_2020_2023_MOESM3_ESM.tif]

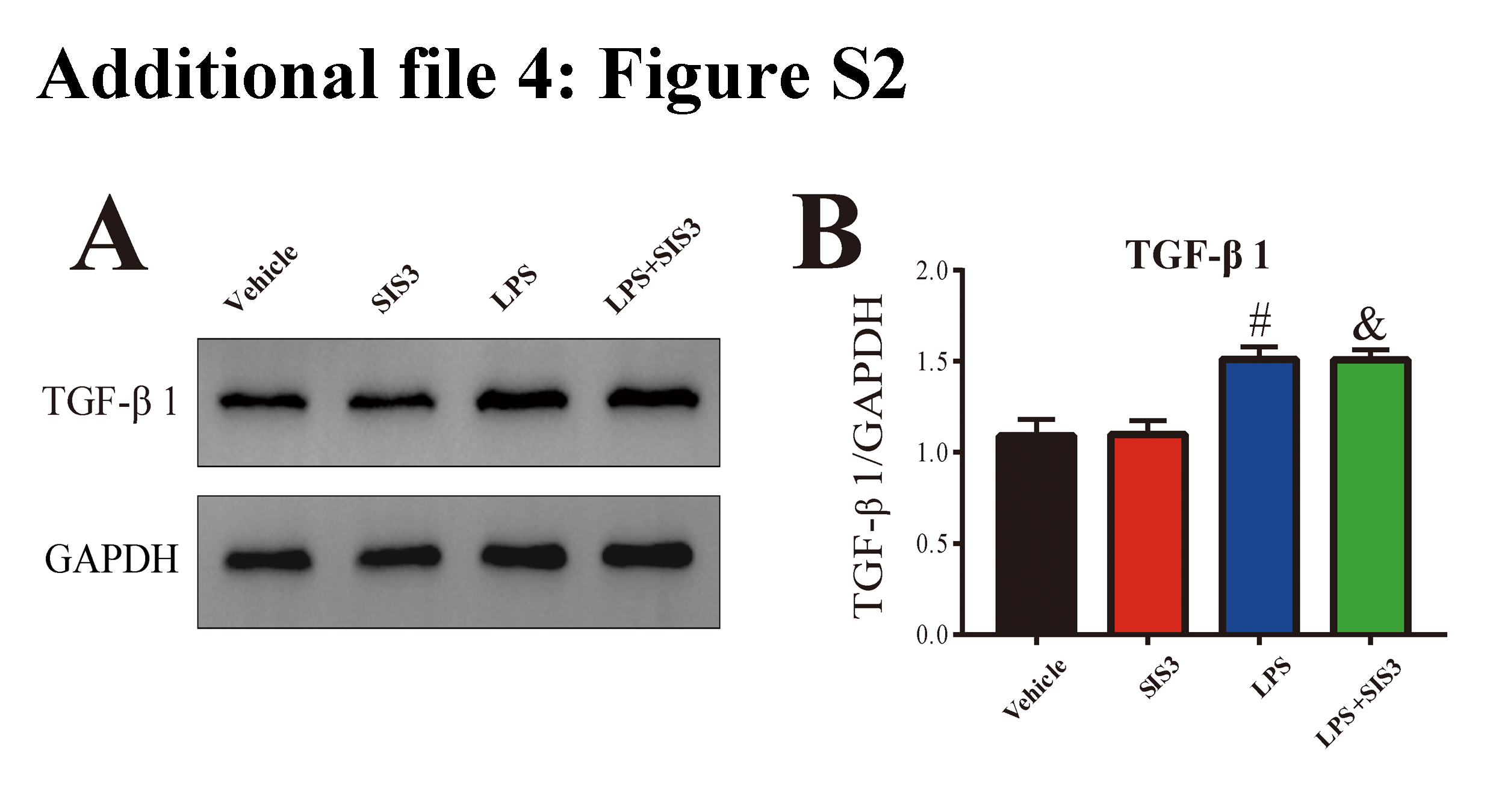

Supplement: Supplementary file 4 — Additional file 4: Figure S2. Effect of SIS3 and LPS on TGF-β1 expression in microglia cultures. (A) Representative figures of TGF-β1 expression (western blotting). (B) Histogram represents quantitation of TGF-β1 normalized to corresponding GAPDH. Results are expressed as mean ± SEM. N = 6. #p < 0.01, compared with the rats treated with vehicle; &p < 0.01, compared with the rats treated with vehicle or SIS3. LPS, Lipopolysaccharide (300 ng/ml); SIS3 (10 μM). [file 12974_2020_2023_MOESM4_ESM.tif]

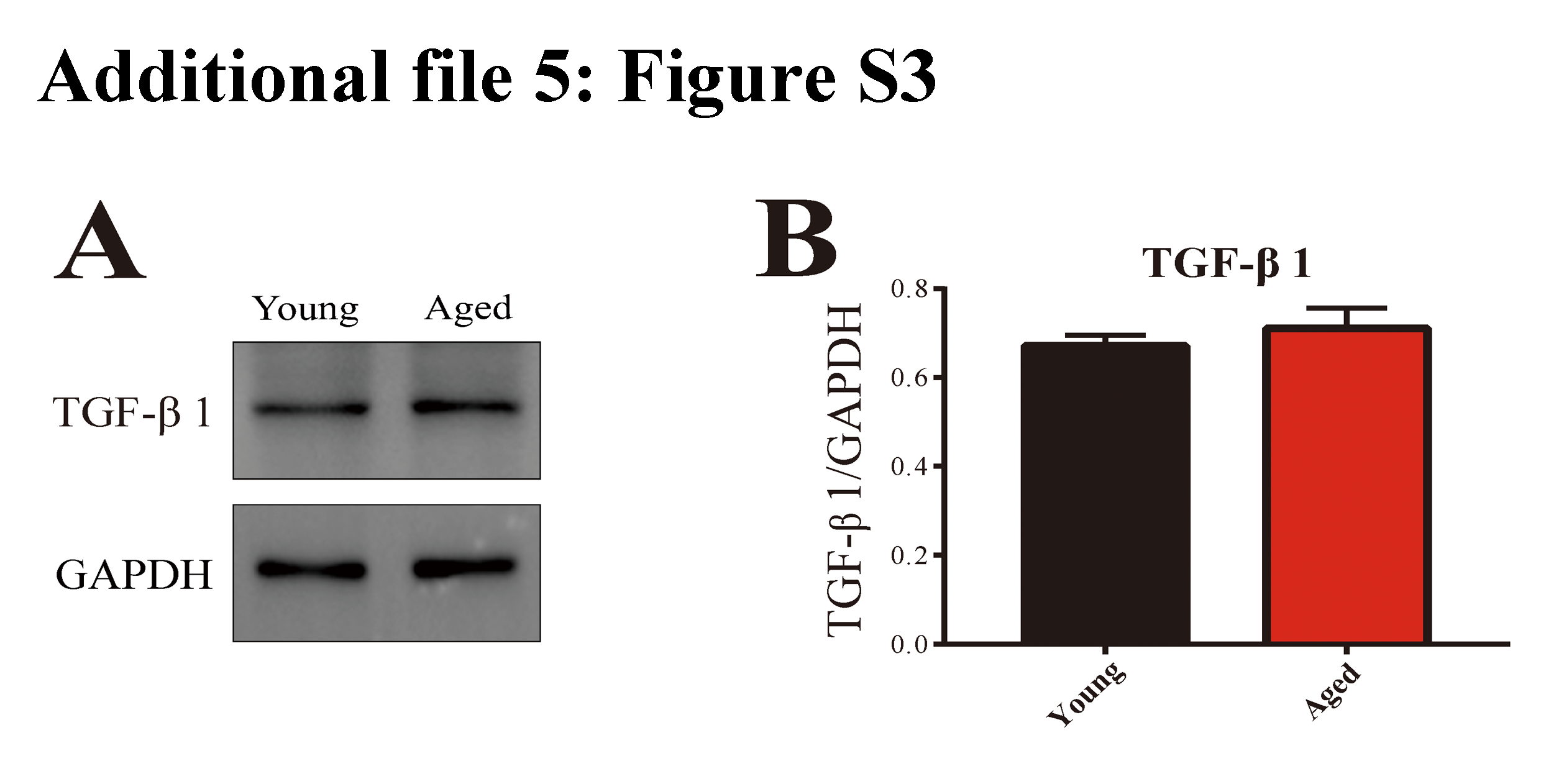

Supplement: Supplementary file 5 — Additional file 5: Figure S3. TGF-β1 expression in the SN of young and aged mice. (A) Representative figures of TGF-β1 expression (western blotting). (B) Histogram represents quantitation of TGF-β1 normalized to corresponding GAPDH. Results are expressed as mean ± SEM. N = 6. [file 12974_2020_2023_MOESM5_ESM.tif]
